# Bioactives from Mushroom: Health Attributes and Food Industry Applications

**DOI:** 10.3390/ma14247640

**Published:** 2021-12-11

**Authors:** Aarti Bains, Prince Chawla, Sawinder Kaur, Agnieszka Najda, Melinda Fogarasi, Szabolcs Fogarasi

**Affiliations:** 1 Department of Biotechnology, CT Institute of Pharmaceutical Sciences, South Campus, Jalandhar 144020, India; aarti05888@gmail.com; 2 Department of Food Technology and Nutrition, Lovely Professional University, Phagwara 144411, India; sawi_raman@yahoo.co.in; 3 Department of Vegetable and Heerbal Crops, University of Life Science in Lublin, 50A Doświadczalna Street, 20-280 Lublin, Poland; agnieszka.najda@up.lublin.pl; 4 Department of Food Engineering, University of Agricultural Sciences and Veterinary Medicine of Cluj-Napoca, Calea Mănăstur 3–5, 400372 Cluj-Napoca, Romania; 5 Department of Chemical Engineering, Faculty of Chemistry and Chemical Engineering, Babeş-Bolyai University, 11 Arany Janos Street, 400028 Cluj-Napoca, Romania

**Keywords:** mushroom, *Trametes versicolor*, bioactivity, health attributes, anti-inflammation

## Abstract

It is well-known that the utilization of mushrooms as therapeutic agents is not new. Over the past years, they have been used by local individuals as food, as well as medicines, throughout the world. Nowadays, mushrooms are excessively used in the medicine, pharmacy, food, and fermentation fields as well. Wild mushrooms are of particular interest, especially *Trametes versicolor* (commonly known as turkey mushrooms) due to their various uses in the food and pharmaceutical industries. They represent not only a huge storehouse of vitamins, minerals, and dietary fiber, but they are also an important source of bioactive polysaccharides. They are widely used in traditional oriental therapies. The fruiting bodies are used in the preparation of health tonics and tea. The present review is necessary to explore more about this mushroom-like classical taxonomy, morphology, nutritional value, bioactivity, various health attributes, mechanism of bioactive components against various diseases, and food applications. The influence of processing processes on the nutritional properties and bioactivity of the fungus is discussed. Potential bioactive components promising health attributes of *Trametes versicolor* are extensively described. Additionally, several in vivo and in vitro studies have demonstrated the beneficial effects of polysaccharopeptides (PSP) and Polysaccharide-K (PSK) on the aspects related to immune function and inflammation, also presenting an anticancerous effect. Moreover, PSP and PSK were successfully described to decrease several life-threatening diseases. The potential food applications of *Trametes versicolor* were detailed to signify the effective utilization of the mushroom in functional food formulation.

## 1. Introduction

Over the past years, wild mushrooms have attained remarkable interest in the fields of medicine and food processing due to their proficient nutritional and therapeutic properties [1,2,3,4]. Globally, about 14,000 species of mushrooms are known, and among them, about 2000 species are considered edible mushrooms [5]. As well, almost 200 species of mushrooms have been commercially cultivated for the formulation of ayurvedic medicine and human consumption [6]. Moreover, wild mushrooms, due to potential nutritional and health attributes, can be compared with various meat, fish, egg, and dairy products [7,8]. Besides nutritional importance, mushrooms are well-known for their bioactive compounds (lectins, polysaccharides (β-glucans), polysaccharide-peptides, polysaccharide-protein complexes, lanostanoids, other terpenoids, alkaloids, sterols, and phenolic structured compounds), which are responsible for different biological and therapeutic activities, including antimicrobial, antioxidant, anti-inflammatory, antidiabetic, anticancerous properties, antiviral, and anti-immunomodulatory activities [9,10,11]. Due to these astonishing properties, mushrooms have been used as key ingredients for the production of many types of functional foods. For instance, there have been made several attempts to incorporate wild edible mushrooms such as *Boletus edulis* and *Agaricus bisporus* into different meet products. Tibulca et al. used *Agaricus bisporus* to enhance the nutritional properties and increase the self-life of pork liver pâté, while Nagy et al. used *Agaricus bisporus* to fortify smoked sausages [11,12]. Other studies reported the efficiency in using *Boletus edulis* to obtain innovative fortified bakery products and beef burgers [13,14]. Among all the wild mushrooms, *Trametes versicolor* (commonly known as turkey tail) mushrooms have attained tremendous popularity due to their broad-spectrum utilization in the food and pharma industries [2]. It is a saprotrophic mushroom species that can be commonly found to grow on dead logs, stumps, tree trunks, and branches throughout the wooded temperate zones of Asia, Europe, and North America. It also represents one of the most common shelf mushrooms in the Northern Hemisphere [8,10]. Although an inedible species, it is traditionally used in Asia as an alternative medicine in the therapy of many diseases, including cancers and certain infections. Morphologically, it is a white-rot lignicolous fungus commonly grown on pine and oak trees, and it is widely utilized as a traditional medicine in various Asian and African countries. However, due to its polypore nature and woody taste, it is less-preferred as food; therefore, its dried powder can be added as an effective ingredient in green tea for its consumption [15]. Furthermore, it is a rich source of dietary fiber and vitamins B1, B2, D2, and C [16]. It can be utilized as a therapeutic component, as it has distinctive medicinal properties due to polyphenols, polysaccharides, proteins, and terpenoids. Functional fiber β-glucans have also been reported in this mushroom that revealed broad-spectrum biological activity. The sterol compounds and terpenoids are responsible for their antioxidant, antimicrobial, and antiviral activity [2]. However, the mushrooms are widely used in traditional therapies; yet, the pharmacological potential of these mushrooms is still not well-explored [17]. Additionally, the aqueous and alcoholic extract of *Trametes versicolor* contains several polysaccharide fractions, including ß-glucan, a D-glucose polymer, along with units of glucuronic acids, arabinose, mannose, fucose, galactose, and xylose, and these polysaccharides are responsible for several biological activities [18]. In contrast, the fruiting body of *Trametes versicolor* is utilized for the preparation of healthy tonics and tea [19]. Polysaccharopeptide Krestin (PSK) and polysaccharopeptides (PSP) are two well-known polysaccharopeptides that are extracted from the mycelial culture of *Trametes versicolor*, and among these two polysaccharopeptides, PSK was produced on a commercial scale by Kureha Chemicals, Iwaki, Japan and ranked 19th among all the commercially successful drugs [20]. About 10 years after the commercialization of PSK, PSP was introduced in the market. In addition to these, there are numerous bioactive compounds isolated from *Trametes versicolor* that are used for traditional medicines and nutraceuticals. The polysaccharopeptides extracted from *Trametes versicolor* are utilized as nutraceuticals preparations in the form of capsules, tablets, syrups, tea, and food additives [21]. Therefore, the present review is necessary to explore more about this mushroom-like classical taxonomy, morphology, nutritional value, bioactivity, various health attributes, mechanism of bioactive components against various diseases, food applications, and the effects of the food processing conditions on the nutritional and bioactive properties of this mushroom.

## 2. Morphological Features of *Trametes versicolor*

**Figure 1:**
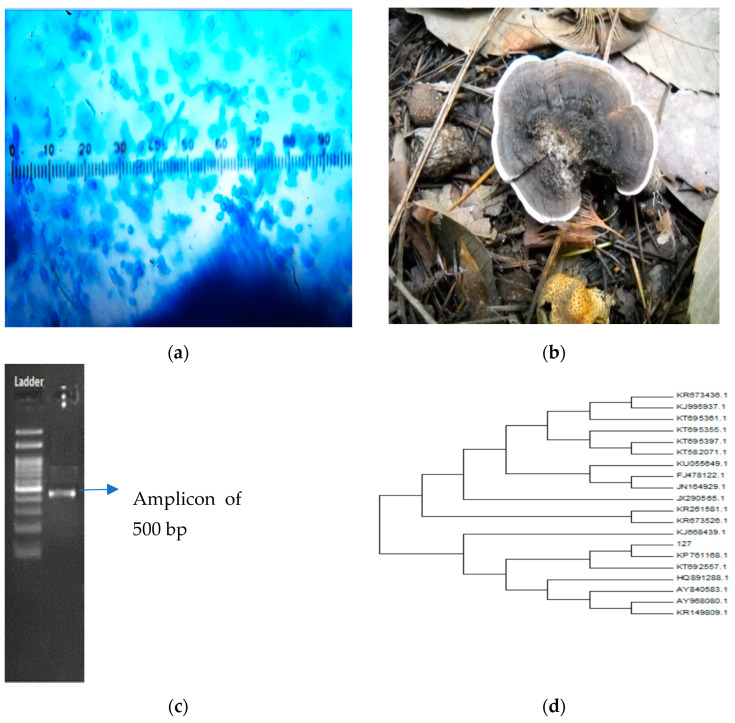
The classical taxonomy of *Trametes versicolor*, which was assigned as sample number 127 when collected from the forest of Solan, Himachal Pradesh, India. (**a**) Fruiting body of sample 127 (*Trametes versicolor*). (**b**) Spore structure. (**c**) The 5.8S rDNA PCR replicon. (**d**) Based upon the phylogenetic analysis, sample 127 showed 98% homology with the published NCBI sequences and identified as a *Trametes versicolor* strain. The nucleotide sequence of the mushroom sample was submitted to NCBI, and it was provided with the GenBank accession number KU 892065.

Classical taxonomy is a traditional method that involves the study of both microscopic and macroscopic traits of mushrooms. *Trametes versicolor* is fan-shaped polypore whose margins are wavy, with colored concentric zones, and belongs to the family Basidiomycotina. These mainly grow on dead wooden logs, trunks, and branches of the tree, and that is considered as an obligate aerobe [22]. The fruiting body of this mushroom consists of a cap that is semicircular, bracket-shaped, fused with concentric zones, smooth brownish-white, pale yellow, grey, or greenish, with a few mm in thickness and 8–10 cm across in length. Furthermore, the pore surface of the mushroom is white to pale grayish, whereas the flesh is insubstantial, tough, leathery, and whitish in appearance [23]. Spores of the mushrooms are smooth, cylindrical, and inamyloid in shape (Figure 1a,b).

Moreover, the life stages of the mushrooms are affected by different physiological alterations that include a change in color, size, and shape; therefore, there is a need for other methods, such as mycelial features and the vegetative growth rate, to differentiate between species and strains [24]. Additionally, DNA-based identification is another method that proved to help in the identification and the taxonomic discrimination of mushrooms. Furthermore, the molecular identification of mushrooms can be done using either the fruiting body or mycelial culture. In this method, ITS (Internal Transcribed Spacer) primers, known as universal primers, are used, which are amplified with the ITS region of two highly variable DNA sequences that are located between a more conserved sequence of large subunit within the rRNA gene and a small subunit 5.8S ribosome (Figure 1c,d). ITS regions have high divergence levels that can easily be amplified, have sufficient type data, are located between highly conserved regions, and generally have a faster rate of evolution in comparison with the nuclear coding region nDNA (nuclear DNA) and DNA present in the mitochondria; hence, it can be used for a phylogenetic relationship analysis, as well as to distinguish the closely related species [25].

## 3. Bioactive Compounds

Medicinal mushrooms, among which many of them belong to the wood-inhabiting, such as polypore, mushroom species can be utilized as an excellent natural source of bioactive components that could have a distinct impact in maintaining human health and prevention from various disease states, expressing various pharmacological effects such as antidiabetic, antitumor, immunomodulating, cardiovascular, antimicrobial, hepatoprotective, and antioxidative [1,2,5,9]. However, their bioactive potentials can be strictly strain-specific, while their chemical compositions are mostly dependent upon specific environmental conditions of their microhabitats—in particular, the physicochemical characteristics of the narrow surroundings of mycelia existence [5,6,7].

*Trametes versicolor* contains a high amount of polysaccharides, including heteroglycan macromolecules, which have a high potential for structural variability. Glucans have been isolated from *Trametes versicolor* and mycelia sources to date, and they are interesting due to having effective therapeutic agents [25]. Additionally, *Trametes versicolor* is known to contain pharmacologically active secondary metabolites that are confirmed through different techniques, including liquid chromatography-mass spectrometry (LCMS), and nuclear magnetic resonance (NMR) spectroscopy. Various reports have been published on the bioactive constituents of *Trametes versicolor*, and scientists have also isolated different phenolic acids, flavonols, flavones, coumarins, isoflavonoids, and bioflavonoids from aqueous, methanolic, and acetone extracts through the LCMS technique [26,27,28]. In this context, Jin et al. isolated 27 compounds from the fruiting body of *Trametes versicolor* and identified them through the NMR spectroscopy technique [29]. The compounds include nine triterpenoids, eight sterols, two ribonucleotides, four phenols, three glycosides, and one feron. In another study by Habibi et al., the chemical properties of the extract of the mushroom prepared in three different solvents: n-hexane, chloroform, and ethyl acetate were evaluated [30]. In their study, they isolated different compounds for the first time; these included five sterols, derivatives of two triterpenes, one hydroquinone-derived aromatic compound, one cerebroside, and one triglyceride derivative. Some other compounds were also isolated in the same study, which included ergosterols, ergosterol peroxide, trilinolein, ergosta 7, 22-dien-3β-ol, and betulin. All these compounds were obtained through thin-layer chromatography and further identified through a NMR spectroscopic analysis. The compounds isolated from the LC-MS and NMR techniques are listed in Supplementary Materials Table S1 and from TLC are listed in Supplementary Materials Table S2.

## 4. Biological Active Components of *Trametes versicolor*

*Trametes versicolor* is composed of minerals, vitamins, and carbohydrates. Polysaccharopeptide Krestin (PSK) and polysaccharopeptides (PSP) are two well-known polysaccharopeptides extracted from the mycelial culture of *Trametes versicolor*. Both PSK and PSP are proteoglycans with a molecular weight of 100 kDa and variations in the compositions of sugars such as glucose, fructose, mannose, galactose, and xylose. These were obtained from COV-1 and CM-101 strains of the mushroom. In the year 1977, in Japan, was the first time PSK was used as an adjuvant for the treatment of gastric cancer patients, and in China, it was used for the first time in 1987 after a series of clinical trials. PSP contains glucans without the peptide that consists of the main chain and branches of β1–3 and β1–6 when extracted extracellularly from the fermentation broth of *Trametes versicolor* [31,32]. PSP extracted intracellularly from the mycelium is a glycopeptide, having peptides that are covalently linked. PSP constitutes the β1–4/β1–3 or β1–4/β1–6-linked backbone of glucose with β1–3 or β1–6-linked branches of glucose [33]. PSK constitutes D-glucose as a major monosaccharide, along with Fru, Gal, Man, and Xyl as the principal monosaccharides. These two proteoglycans obtained from mushrooms have medicinal importance and, therefore, are considered to be the most promising compounds. Among them, PSP has been considered as a wide spectrum response modifier. Moreover, PSP has a low cytotoxicity and acts as an immunomodulating and anticancerous agent; therefore, it has been used to stimulate the immunological conditions of patients under clinical treatment, including both radio- and chemotherapy, and PSP also exhibits analgesic antiviral activity [32].

## 5. Health Attributes of *Trametes versicolor*

Several bioactive compounds are present in the mushroom, and they attribute effective therapeutic and medicinal values to humans. Traditionally, *Trametes versicolor* has been consumed for its potential health attributes, and researchers have explored the health attributes of the mushroom using several in vitro, in vivo, and chemical methods [31,34]. Herein, the bioactive components responsible for the therapeutic importance are described.

### 5.1. Role of Polysaccharopeptides as Prebiotics

**Figure 2:**
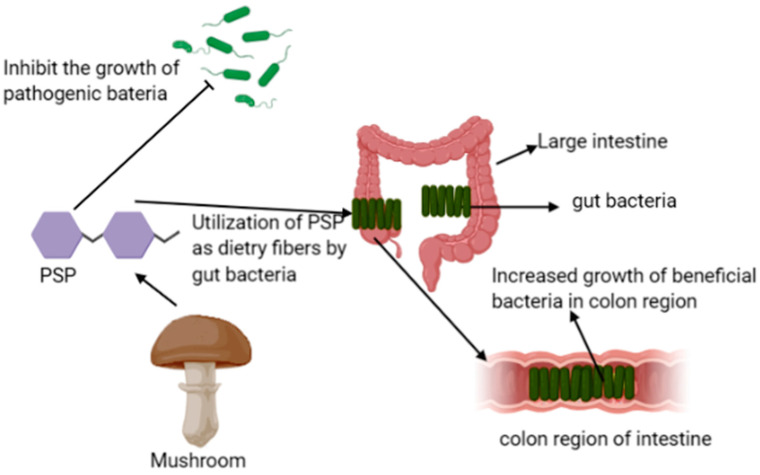
Possible mechanism of PSP of *Trametes versicolor* as prebiotics.

Prebiotics are nondigestible ingredients of food that affect the host beneficially by promoting the growth of beneficial bacteria of the colon and improves the immunity of the host, and the intake of mushrooms as food enhances the growth of probiotic bacteria in the large intestine; therefore, it protects the human body from viral infections and inhibits the growth of disease-causing bacteria such as *E. coli*, *Clostridium,* and *Salmonella* (Figure 2) [34,35].

Edible mushrooms consist of pleuran, lentinan, schizophyllan, α and β-glucans, mannans, chitin, hemicellulose, galactans, and xylans polysaccharides that possess prebiotic effects. These polysaccharides increase the resistance to the intestinal mucosa and ulcer formation in the intestines of rats. In this regard, Yu et al. reported the effects of polysaccharopeptides (PSP) isolated from *Trametes versicolor* on the growth and activity of the probiotic bacteria *Bifidobacteria* and *Lactobacilli* [36]. The polysaccharides of *Trametes versicolor* possess different chemical constituents, including β-glucans as the principal components that are not easily digested by the intestinal enzymes. These polysaccharides, due to their non-starch and nondigestible natures, can be utilized as dietary fibers by gut probiotic bacteria in the large intestine and result in increased growth of these bacteria in the colon region, thereby providing health benefits, and are considered as potential prebiotics [35,37]. However, complete information regarding the ability of β-glycans of mushrooms to inhibit the composition of fecal microbes has not been investigated.

### 5.2. Antidiabetic and Antiobesity Properties

Type 2 diabetes results in chronic diseases, including diabetic nephropathy (disease related to the kidney), diabetic retinopathy (disease related to eyes), and diabetic neuropathy (nontraumatic lower-limb amputations). Due to continuous changes in the eating behavior and activity level of human beings, the incidence of type 2 diabetes is increasing continuously [38]. The disease results due to impaired secretion and resistance of insulin inside the body. The major risk effect for insulin resistance is obesity, and a strong relationship between these two factors has been reported in both animal and human studies [39]. Therefore, improvements in obesity result in the improvement of insulin sensitivity and the prevention of type 2 diabetes. Ternatin is a highly methylated cyclic heptapeptide.

**Figure 3:**
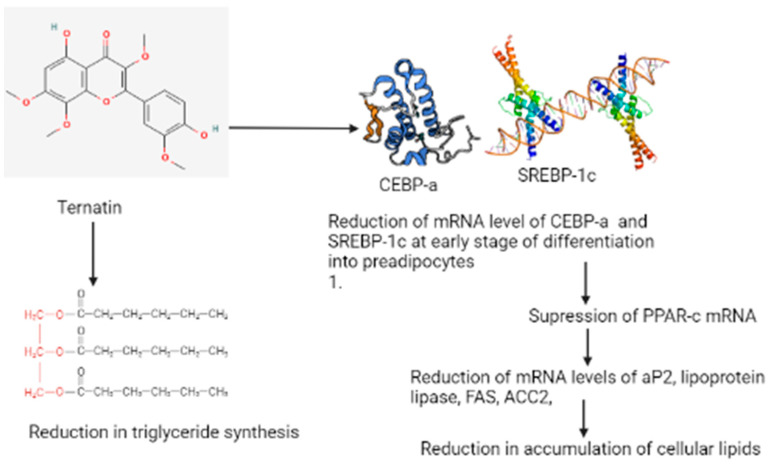
Possible mechanism of the antidiabetic and antiobesity properties of the ternatin compound isolated from *Trametes versicolor*.

This compound inhibits the effect of fat accumulation, which was studied using 3T3-L1 adipocytes [40]. According to Figure 3, ternatin reduced the mRNA levels of CCAAT/enhancer-binding protein-a (C/EBP-a and sterol regulatory element-binding protein-1c (SREBP-1c)) at an early stage of differentiation in preadipocytes; this results in the suppression of peroxisome proliferative-activated receptors c (PPAR-cmRNA) [41]. The suppression results in the reduction of messenger RNA levels of adipocyte fatty acid-binding protein (aP2), lipoprotein lipase, FAS (fatty acid synthase), and ACC2 (acetyl-CoA carboxylase) due to the cellular lipid accumulation becoming reduced [42]. The consumption of ternatin also reduced triglyceride synthesis, but its effectiveness is lower in the differentiation of adipocytes than observed in preadipocytes. D-Leu7 is a derivative of ternatin that is demonstrated to inhibit the accumulation of fats in 3T3-L1 cells and is eight times more effective with a 12-fold higher cytotoxic activity (IC_50_) than that of ternatin [43]. Kobayashi et al., in their study, reported that [D-Leu7] ternatin acts by suppressing the expression of lipogenic genes in diabetic Kuo Kondo yellow obese (KK-A(y)) mice and inhibits the accumulation of fats in cultured adipocytes [44]. They also reported that ternatin and its derivative compound can be a valuable drug for the treatment of obesity. The finding of this study proved that the compound present in mushrooms significantly (*p* < 0.05) lowers the glucose and triglycerides. Furthermore, Xian et al. reported the effect of an aqueous extract of mushrooms in improving the insulin resistance with the Pl3K/Akt and p38 MAPK signaling pathways, which are involved in diabetic skeletal muscles [45]. In their study, they induced insulin-resistant rat models by T2DM with HFD feeding and STZ injection for the manifestation of serious metabolic diseases that exhibit the characteristics of hyperglycemia, hypertension, and dyslipidemia. The insulin-resistant rats in their study displayed certain changes in their bodies that included an increase in venous blood glucose and physiological indexes like the body weight, intake of water, and intake of food. When these rats were treated with mushroom extract in different concentrations of the dosage, the physiological index and glucose level improved compared to the untreated T2DM. In their experiment, it was revealed for the first time that a direct demonstration of the mushroom extract could improve the level of glucose in skeletal muscles. The antiobesity effect of the polysaccharides obtained from *Trametes versicolor* was investigated by in vivo methods, and it was observed that the polysaccharides triggered the splenocytes of mice via the MAPK-NF-κB signaling pathway that induced an immunomodulatory effect [46].

### 5.3. Anti-Inflammatory Properties

**Figure 4:**
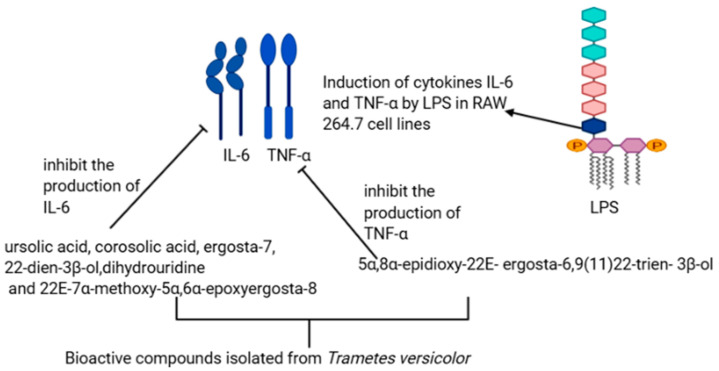
Possible mechanism for the anti-inflammatory activity of bioactive compounds isolated from the extract of *Trametes versicolor*.

Inflammation is, although a defensive phenomenon in the body with insufficient regulation and unsuitable in the disparity of self-tissue, could be the reason for severe diseases and injuries [47]. *Trametes versicolor* consists of bioactive compounds, including terpenoids and sterols, with anti-inflammatory properties. The effects of these compounds were evaluated primarily on the secretion of cytokines nitric oxide, TNF α, and IL-6 in RAW 264.7 cells by Jin et al. [29]. In their study, they found that LPS induced the production of tumor-associated genes cytokines TNF α and IL-6 in RAW 264.7. The cytokines TNF α and IL-6 are inflammatory mediators and are responsible for inflammation. The activity of these mediators was suppressed by the bioactive compounds obtained from *Trametes versicolor*. For instance, 5α,8α-epidioxy-22*E*-ergosta-6 suppress the production of TNF α induced by LPS, while ursolic acid, corosolic acids, 2*E*-7α-methoxy-5α,6α-epoxyergosta-8, ergosta-7,22-dien-3β-ol, and dihydrouridine showed an inhibitory effect of IL-6 induced by LPS (Figure 4). Furthermore, Kojic et al., in their study, treated grape pomace with *Trametes versicolor* for 15 days under solid-state fermentation (SSF) to enhance the anti-inflammatory properties, and they observed an increase of flavan-3-ol and the flavonol rutin within 1–4 days of treatment [48].

The SSF of grape pomace with mushroom increased the inhibition of enzymes 5-lipoxygenase and hyaluronidase that are involved in the inflammatory process resulting in the lowest IC_50_ values of 37 and 444 μg/mL, respectively, on the second and third days of fermentation.

## 6. Bioactivity of *Trametes versicolor* Mushroom Extract

### 6.1. Anticancerous and Immunomodulatory Activity

Polysaccharides of *Trametes versicolor* consist of β-glucans as major components that consist of immune receptors dectin-1, TLRs-2, 4, and 6 and a complement receptor (CR3). These immune receptors are expressed by the activation of a group of immune cells that includes monocytes, natural killer cells, macrophages, neutrophils, and dendritic cells. Therefore, β-glucans directly or indirectly modulate the response of both innate and adaptive immunity [49]. Polysaccharide PSK helps to improve the immunosuppressive state in cancer patients and restore the response of the immune system and antitumor activity. PSK stimulates the response for Toll-like receptors, mainly TLR-2 and TLR-4, and mediate the cell signaling pathways [50]. Additionally, PSP stimulates the response of TLR4 and its downstream molecule TRAF6 and is accompanied when there is an increase in the phosphorylation of the NF-κB p65 transcription factor and c-Jun peritoneal macrophages of TLR4 + mice [51]. There are about 30% of the peptides present in both PSK and PSP that might contribute to the antitumor activity. The peptides of PSP show antitumor activity by the production of antibodies collaborated by both B and T cells. The collaboration of B and T cells showed that relevant amounts of B and T-cell epitopes are contained by peptide–polysaccharide; these epitopes induce high titers of anti-polysaccharide antibodies and antipeptide with an anamnestic response for both polysaccharides and peptides and are characterized by the switching of the immunoglobulin from IgM to IgG. Therefore, in improving the anti-polysaccharide-induced immune response, the peptides with relevant B- and T-cell epitopes act as good carriers. The tumor cells have abnormal cell surface glycan structures to which either peptides of PSP help to generate surface glycan antibodies or the cell surface glycans are cross-reacted with PSP antibodies that contribute to the antitumor activity of PSP [52]. PSP suppresses the proliferation of activated T cells in a suppressive action involved in the MAPKp38 and STAT5 pathways. PSP suppresses the expression of cytokines Th1 interleukins 1β and Interleukin-2, Tumor necrosis factor α, (TNF α), and IFN-I in a time-dependent manner but fails to suppress the expression of Th2 cytokines IL-4 and IL-10 [53]. PSP induces a reduction of the CD3+/CD5+ cells that helps the cytokines’ balance shift towards Th2 dominance; therefore, PSP has several direct and indirect cellular target cancer and immune cells and can stimulate both T cells and macrophages [54]. PSP initiates both acquired and innate immunity in vivo, as it activates macrophages, natural killer cells, and T cells and also stimulates the secretion of TMF, several interleukins, and reactive nitrogen and oxygen intermediates [55].

#### Mechanism of Antitumor and Immunomodulatory Effects of PSP

**Figure 5:**
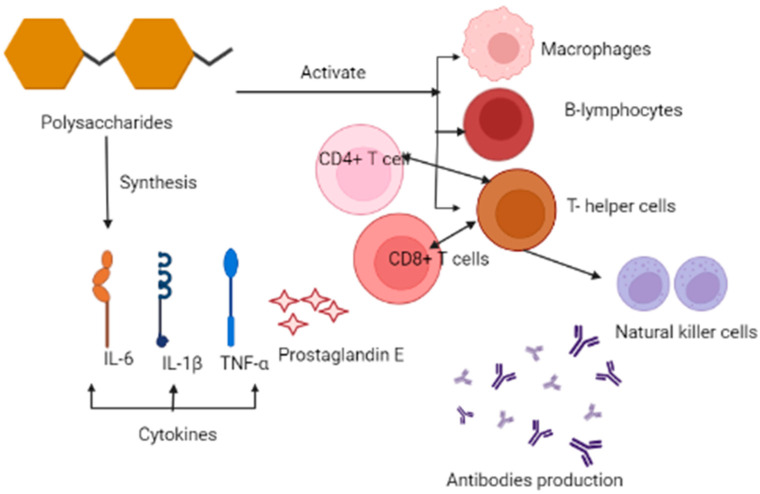
Mechanism of the anticancerous and immunomodulatory effects of PSP of *Trametes versicolor*.

PSP regulates the function of the immune system by activating macrophages, B-lymphocytes, and T-lymphocytes and promotes the formation of antibodies, as well as activation of the complement system (Figure 5).

PSP reduces cancer treatment-related adverse side effects that include a loss of appetite, vomiting, pain, and fatigue by antagonizing the decline of function of the immune cells that result from the tumor itself. PSP causes the synthesis of cytokines interleukin-1β, IL-6, TNF α (tumor necrosis factor α), PGE2 (prostaglandin E2), ROS (reactive oxygen species), and nitrogen mediators. These polysaccharides also prohibit the proliferation of cancer cells both in vivo and in vitro [51].

### 6.2. Antiviral Activity

The study of the antiviral activity of mushrooms was started in the second half of the 20th century and was reported for the first time from an extract of the mushroom *Lentinula edodes* [49]. In 1977, the Ministry of Japan approved the polysaccharides (PSK) fraction obtained from *Trametes versicolor*, and by 1987, the fraction accounted for 25.2% of the total expenditure for the manufacturing of anticancerous agents [56]. In contrast, Liu et al. revealed that PSK can inhibit the growth of B cells and activate natural killer cells and T cells in EBV (Epstein–Barr virus)-infected blood lymphocytes of the umbilical cord and exert increased cytotoxicity against B cells infected with EBV [57]. Furthermore, Ng et al. reported that, in a clinical trial, the food additive of the mushrooms reduced the frequency and inhibited the effect of the HSV-2 virus in pregnant women completely [58]. Additionally, Ibragimova et al. revealed the effect of the extract of the fruiting body of *Trametes **versicolor*** on the influenza virus in vivo and observed a slight inhibition of influenza virus A/Chicken/Kurgan/05/2005 (H5N1) [59]. In another study by Teplyakova et al., the effect of an aqueous extract of *Trametes versicolor* 2263 mycelium was observed on MDCK cells to repress the virus H5N1 (A/Chicken/Kurgan/05/2005) (2.251 g) H3N2 (A/Achi/2/68) (0.51 g) [60]. The antiviral properties of the mycelial culture of *Trametes versicolor* 353 were evaluated against the A/FM/1/47 strain of HSV1 (Herpes simplex virus type 1) and the HSV 2 (Herpes simplex virus type 2) strain BH on MDCK cells and RK-13 cells [61]. It has been observed that the mushroom inhibits HSV-2 replication in RK-13 cells, which have a higher therapeutic index (342.67). The polysaccharides PSP and PSK reported from *Trametes versicolor* showed inhibitory effects on HIV (Human immunodeficiency virus) in vitro. The polysaccharide showed an immune-stimulatory effect, Krestin from PSK supported the killer cells of the immune system, the PSP complex the activity of HIV reverse transcriptase, and prohibited HIV-1 gp120 attachment to the CD4 surface receptor. PSP activates the TLR4-signaling cascades, thereby stimulating the immune system, which results in the production of cytokines and chemokines [62,63]. In another study by Rodríguez-Valentín et al., the TLR-dependent immune response was determined to check the efficacy of PSP isolated from *Trametes versicolor* as an anti-HIV agent [64].

#### Mechanisms of Action of PSP as Antiviral Agents

**Figure 6:**
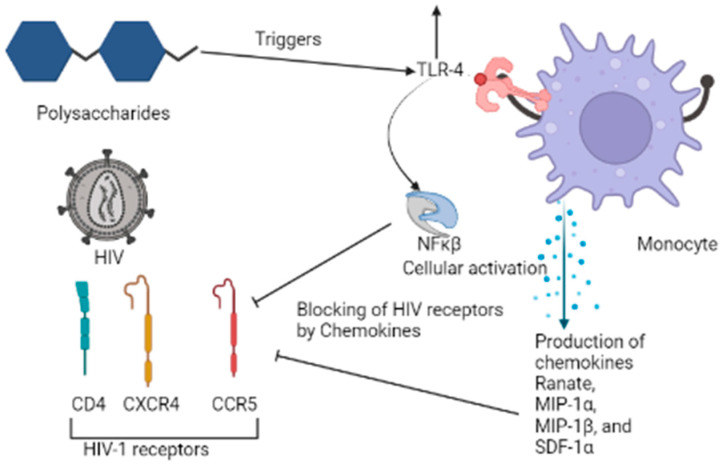
Mechanisms of action of PSP of *Trametes versicolor* on the HIV-1 virus.

PSP obtained from *Trametes versicolor* can trigger Toll-like receptor-4 (TLR-4), which is the most potent receptor that counters the HIV infection in macrophages present inside the human body (Figure 6).

It leads to the activation of NF-κβ and antiviral chemokines RANTES, MIP-1α, MIP-1β, and SDF-1α. These antiviral chemokines block the HIV-1 receptors CCR5 and CXCR4, which results in the subsequent downregulation of the replication of HIV [64].

### 6.3. The Antiparasitic Activity of Trametes versicolor

Parasites are the organisms living in the host to get food from or at the expense of [65]. They can cause disease in humans; parasites infect human beings, include protozoa and worms, and are classified as endoparasites, causing an infection inside the body, and ectoparasites, causing disease superficially within the skin. *Leishmania* is one of the protozoa that causes leishmaniasis, a widespread tropical and subtropical disease. The genus *Leishmania* is transmitted by insects, and the reservoir of the parasites are domestic animals like dogs, rabbits, and small mammals. The parasite invades different organs of the body, causing harsh injuries or even death. Many compounds are isolated from plants, and drugs are used to fight against the infection caused by the parasite, but due to the high toxicity of drugs and resistance of the parasite, it becomes difficult to cure the infection [66]. The illness caused by the parasites increases in the number of infected persons more than 1.5 million annually; therefore, it requires natural products that offer promising sources of chemical diversity for the development of new drugs [67]. *Trametes versicolor* constitutes various bioactive compounds that exhibit different biological activities, among them trametenolic acid, considered as a promising compound responsible for anti-leishmanial activity [68]. Furthermore, Chan-Bacab et al. and Leliebre-Lara et al. isolated three sterols compound named 5α, 8αepidioxy-22E-ergosta-6, 5 αergost-7, 22-dien-3 β-ol, and 3 β hydroxylanostan-8, 24-diene-21-oic acids (trametenolic acid) from the hexane extract prepared from fruiting bodies of *Trametes versicolor* [69,70]. All these sterol compounds were evaluated for their anti-leishmaniasis activity and observed that, among these three sterol compounds, 3 β hydroxylanostan-8, 24-diene-21-oic acids (trametenolic acid) showed the highest activity against both the intracellular and extracellular stages of *Leishmania amazonesis* and therefore proved to be an excellent source for the discovery of the new leishmanicidal drug [70]. The mechanism of action of the particular compound is yet not known, and more study is required to elucidate its mechanism of action.

### 6.4. Antimicrobial Activity of Trametes versicolor

In the recent era, resistance to pathogenic microorganisms against different antibiotics has become a global concern. Both Gram-positive and Gram-negative bacteria have different mechanisms against antimicrobial agents, and there is a need to discover a new and effective alternative against these microorganisms [71,72]. Mushrooms constitute different bioactive compounds that express high antimicrobial activity against both Gram-positive and Gram-negative bacteria. Several studies showed that *Trametes versicolor* has effective results against several diseases causing pathogenic microorganisms [73]. Furthermore, Ng et al. did in vivo animal studies to evaluate the antimicrobial activity of *Trametes versicolor* against *E. coli*, *P. aeruginosa*, *S. aureus*, *Candida albicans*, *Klebsiella pneumoniae*, *L. monocytogenes*, and *Streptococcus pneumonia* [74]. In another study by Karaman et al., it was observed that the methanol extract of *Trametes versicolor* showed antimicrobial activity against *S. aureus* and *Bacillus* sp., while Gram-negative bacteria *E. coli*, *S. flexneri*, and *P. mirabilis* were resistant [75]. Alves et al. isolated compounds sesquiterpene and coriolan that inhibited the growth of Gram-positive bacteria [73]. In addition to this, they also tested the antimicrobial activity of phenolic compounds protocatechuic and caffeic acid against *S. aureus*, *S. epidermidis*, and *L. monocytogenes* with a MIC of 1mg/mL. Yamaç et al. observed a weak effect (<10 mm zone of inhibition) of acetone extract prepared from the mushroom against *P. aeruginosa* [76]. In this context, Özkök et al. studied the effect of chloroform extract prepared from *Trametes versicolor* and observed that the mushroom has effective results against *S. aureus* and *E. faecalis* [77]. The difference in sensitivity among strains of bacteria is due to the presence of the outer membrane of Gram-negative bacteria that surrounds the peptidoglycan layer and restricts the diffusion of LPS (lipopolysaccharide). The LPS provides selective permeability and serves as a barrier to the penetration of harmful agents and compounds. On the other hand, Gram-positive bacteria lack an outer membrane, having a structure that is porous, thick, and hydrophilic; this structure makes it permeable and allows the entry of compounds from outside [78,79].

### 6.5. Trametes versicolor Uses in The Food Industry

*Trametes versicolor* possess a wide range of both intracellular and extracellular enzymes, which are responsible for producing a broad spectrum of natural flavor compounds [80]. *Trametes versicolor* with a fermentation time of 38 h produces a pleasant aroma profile. *Trametes versicolor* produce protein-bound polysaccharides, which are used in the preparation of different kinds of promising dietary supplements. These supplements are marketed in powder form, tablets, and capsules and, hence, also used for the production of nonalcoholic cereal-based beverages. In a study conducted by Zhang et al., the wort was fermented by *Trametes versicolor* to develop a nonalcoholic or low-alcoholic beer for the first time [81]. In their study, they observed that during fermentation there was a reduction of total reducing sugar and glucose by 21% and 43%, respectively. The concentrations of the proteins reduced about 5%, and the oxalic acid concentration, which is present in wort, reduced from 28 to 23mg/mL. The oxalic acid was converted to formic acid and carbon dioxide by the enzyme oxalate decarboxylase produced by *Trametes versicolor*. The concentration of ethanol in the fermented beverage was 0.39%, whereas, in wort, no ethanol was detected. After the production of ethanol, the product was analyzed for its flavor, an analysis was done by the GC-MS/ MS-O method, and a total of 28 flavoring compounds were identified, as listed in the Supplementary Materials (Table S3). In another study conducted by Dhillon et al., a laccase enzyme was produced by the mushroom *Trametes versicolor* cultured in brewer’s spent grain for the removal of haziness and flocculation from crude beer [82]. In their study, the flocculation properties of the crude beer were studied using different parameters that included viscosity, turbidity, total polyphenols, and the protein content, and it was observed that the treatment with laccase showed a good flocculation capacity in comparison to the industrial flocculation process and, therefore, can be used as an alternate to stabifixin and benoti, the traditional flocculants. In this context, Takemori et al. used crude laccase isolated from *Trametes versicolor* to enhance the flavor and taste of cacao nib and its products, and it was observed that, upon treatment with laccase, the bitterness and other unpleasant tastes of the products are removed completely and taste better than the control [83]. Furthermore, Di Fusco et al. developed an amperometric biosensor for the determination of polyphenol index in wines and its performance was dependent on laccases present in *Trametes versicolor* and *Trametes hirsuta* [84]. In another study by Ibarra-Escutia et al. amperometric biosensor-based laccase from *Trametes versicolor* was developed to monitor the phenolic compounds in tea infusions [85]. They observed in their study that in terms of response time, operation stability, sensitivity, and process of manufacturing these biosensors showed good results, therefore, without doing any pre-treatment the samples can be used directly to determine the accurate amount of phenolic compounds. Also, Attanasio et al. revealed the technology of nonisothermal bioreactors in the treatment of oil meal wastewater [86]. The isothermal bioreactors with laccase obtained from *Trametes versicolor* were immobilized on the nylon membrane for the detoxification of oil meal wastewater.

## 7. Safety Assessment of *Trametes versicolor* as Food Supplements

Mushrooms are considered functional food that can be consumed as dietary supplements. The nutrients derived from biomass of *Trametes versicolor* are used as supplements consumed in liquid or capsule form, sol-gel, gel caps liquid, or powdered form. In this context, Barros et al. first time revealed a safety assessment of aqueous extract of mushroom biomass on both male and female rats [87]. These animals were administered with varying concentrations of extract (2.5 g, 5.0 g, and 7.5 g/kg live weight/day) for 90 days and observed the appearance of behavior and they found no significant difference between relative organ weights, no changes occur neurologically and hematologically. *Trametes versicolor* is known to be the most commonly used medicinal product in China and Japan and very rare information regarding its toxicological behavior is available, either from biomass form and extract [88]. Additionally, Hor et al. standardized water extract to evaluate the potential toxicity of mushrooms in rats after 28 days and did not found any signs of toxicity [89]. In an accumulating by different researchers, it was found that PSP extracted from the extract is non-toxic when administered with an effective dosage. The PSP when used over extended periods even 100 folds than normal clinical dose also not induced any kind of acute and chronic toxicity in animals and therefore, is not teratogenic [90]. The adverse effect of PSP was also observed in pregnant mice and it was found it did not cause any harmful effect on reproductive and embryonic development in mice and hence appeared to be safe during pregnancy [91]. In another study, Chu et al. calculated LD 50 as 18 g/kg after 90-day administration using an aqueous extract of *Trametes versicolor* having high concentrations than its biomass [92]. According to the classification of Loomis and Hayes a substance having an LD50 value between 5 g/kg and 15 g/kg is considered to be virtually non-toxic [93]. An acceptable intake of biomass in the case of the human being can be established at 4.5 g with an average weight of 60 kg [87].

## 8. Conclusions and Future Perspectives

Wild mushrooms are considered as future food with various health, nutritional, and functional attributes. *Trametes versicolor* mushroom is well known for its bioactive compounds that are responsible for different biological and therapeutic activities including antimicrobial, antioxidant, anti-inflammatory, anti-diabetic, and anti-cancerous properties. Every part of this mushroom contains vital components and is used as an active ingredient for the formulation of various folk medicines and food products. Due to its ability to produce intracellular and extracellular enzymes, it can be used in food industries as a flavoring compound. In addition to this, the fruiting body of mushrooms can be used to manufacture dietary supplements either in powdered form, tablets, or capsules. The mushroom extract has effective antiviral, antibacterial, antifungal, and anti-inflammatory activity, therefore, it could be used as an active ingredient for the preparation of antimicrobial hand sanitizer and anti-inflammatory drugs. However, for future perspectives, more emphasis is required for the formulation of various functional foods for the direct consumption of this mushroom, as no food product is existing to date for the direct consumption of *Trametes versicolor*. As well, more precise toxicity studies are required for the wide utilization of the mushroom. Due to high nutritional and functional values, it could be used for the formulation of space food and for people who are living in highly remote areas. Food authorities should promote more research on mushrooms to develop regulations on mushroom-based products. According to the current situation of the world, due to high bioactivity of the mushroom, it could be used for the development of antiviral drugs.

## Data Availability

Data sharing is not applicable to this article.

## Supplementary Materials

The following are available online at https://www.mdpi.com/article/10.3390/ma14247640/s1: Table S1: Phenolic compounds of *Trametes versicolor* identified through the LC-MS and NMR techniques. Table S2: Phenolic compounds of *Trametes Versicolor* identified through the TLC technique. Table S3: Flavoring compounds isolated from *Trametes versicolor* by the GC-MS/MS-O method.

## Abbreviations

PSK	Polysaccharopeptide Krestin
PSP	Polysaccharopeptide
ITS	Internal Transcribed Spacer
SSF	Solid-state fermentation
