# Supplementary material for: Bioactives from Mushroom: Health Attributes and Food Industry Applications
Source: Materials (Basel). 2021 Dec 11;14(24):7640. doi: 10.3390/ma14247640 (PMC8706457; doi:10.3390/ma14247640)
Supplement: Supplementary file 1 [file materials-14-07640-s001.zip › materials-1463069-supplementary.pdf]

Table S1. Phenolic compounds of Trametes versicolor identified through LC-MS and NMR technique

Compound Isolated	Molecular structure	References
Quinic acid	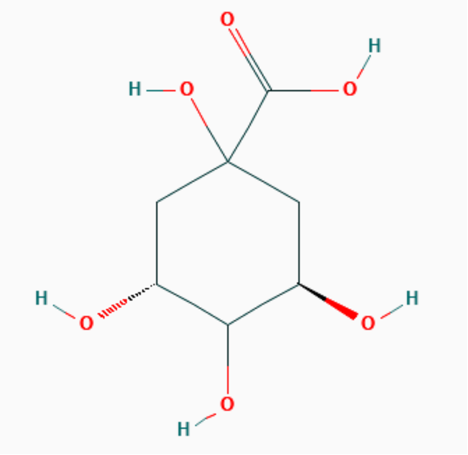	
p-Coumaric acid	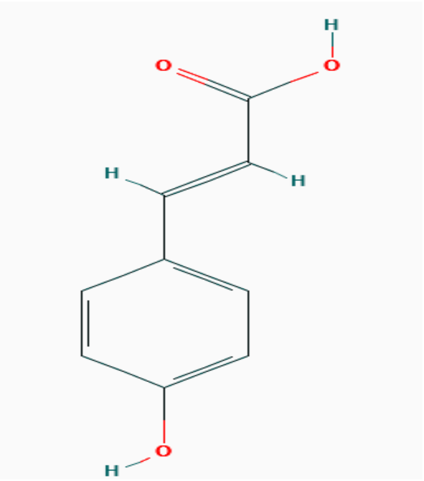	[23]
Malic acid	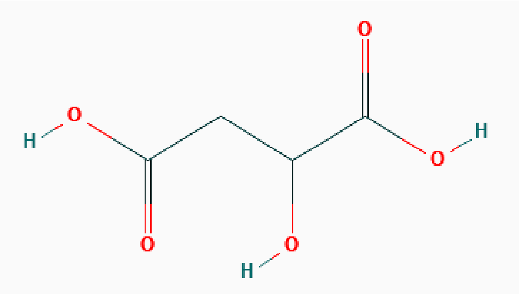	

Caffeic acid	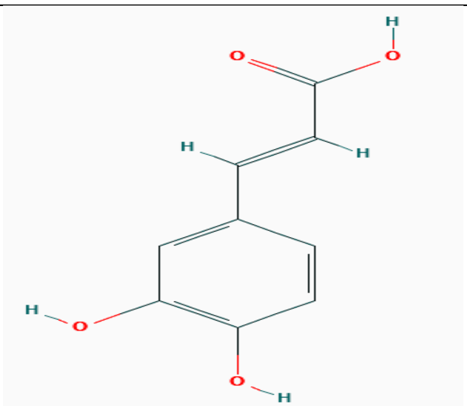
p-Hydroxybenzoic acid	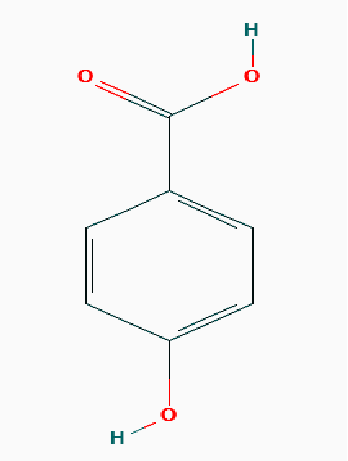
Vanillic acid	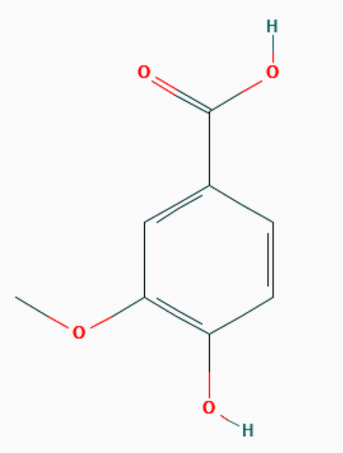
Syringic acid	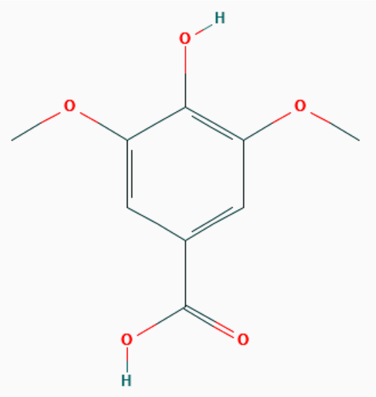

Gallic acid	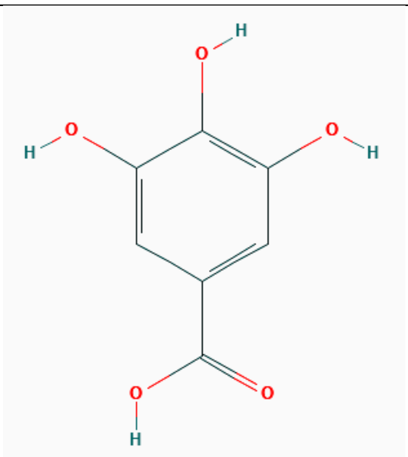
Ferulic acid	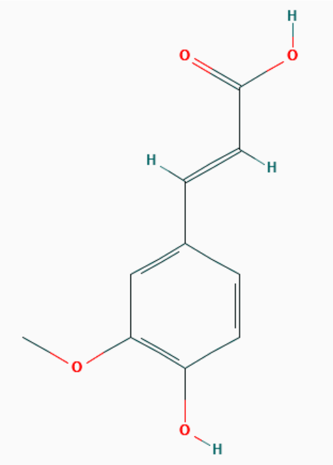
Quercetin - glucuronide	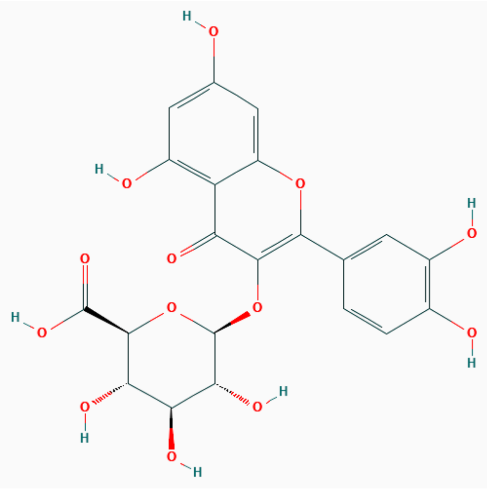

Rutin	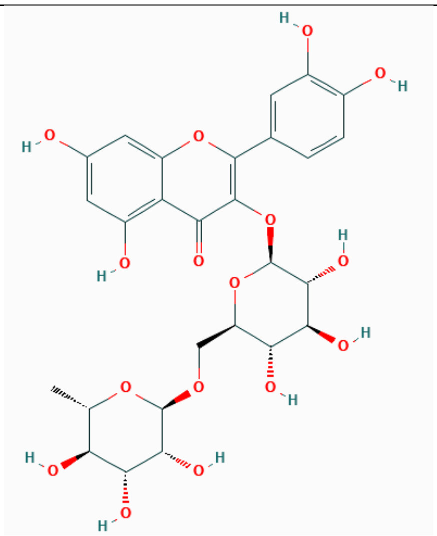
Quercetin	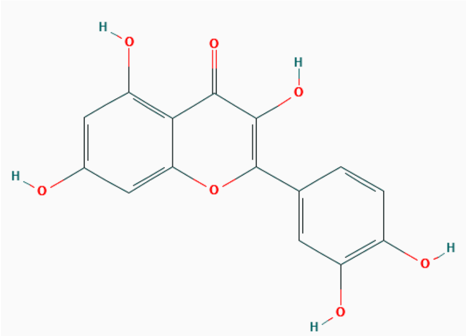
Baicalein	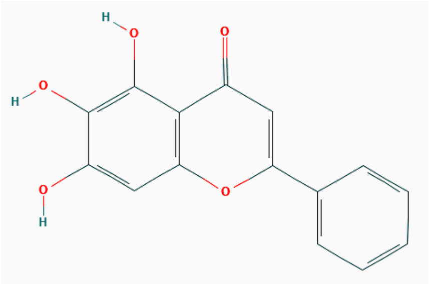
Apiin	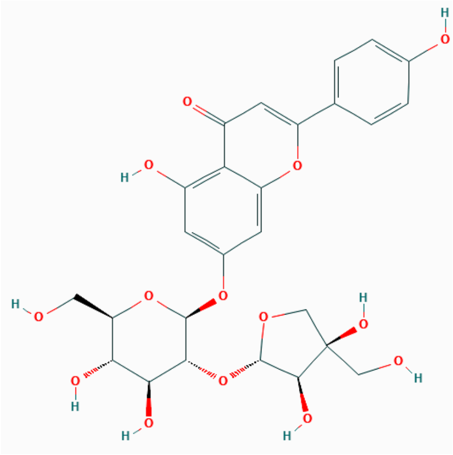

Vitexin	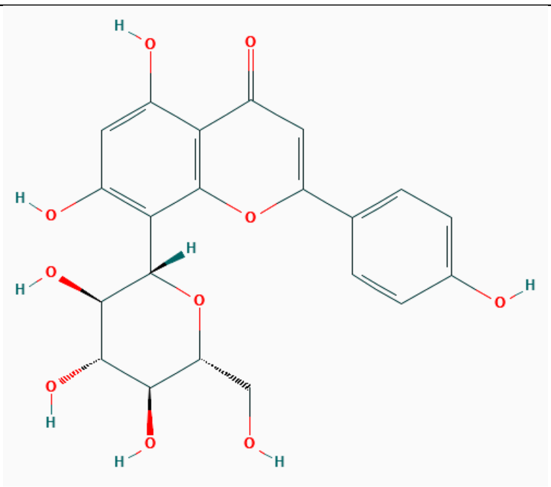
Apigenin	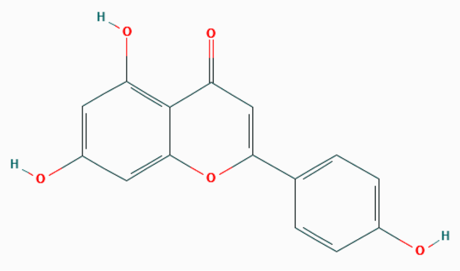
Catechin	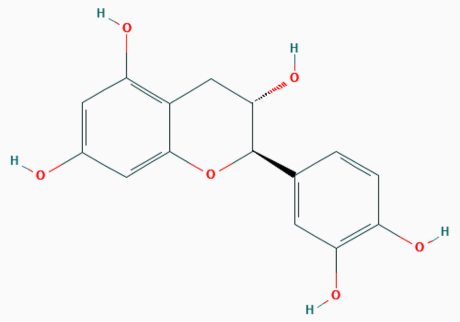
Daidzein	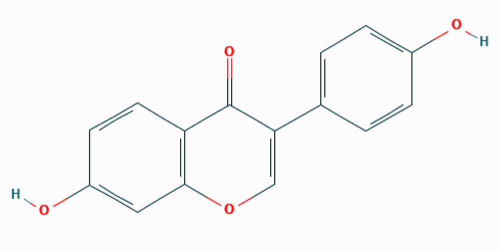

Amentoflavone	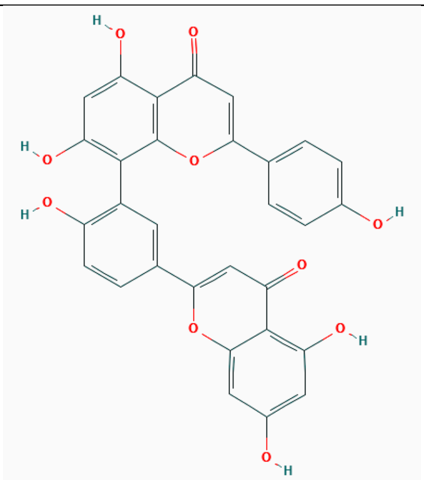	
Friedelin	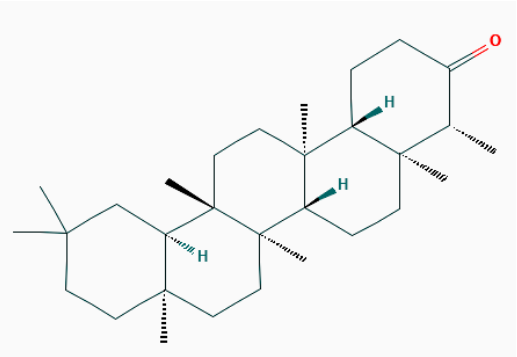	
Glutinol	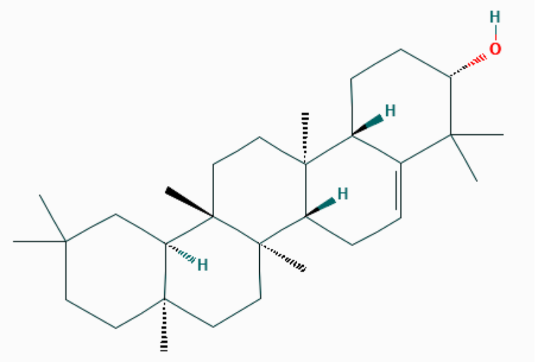	[24]
Ursolic acid	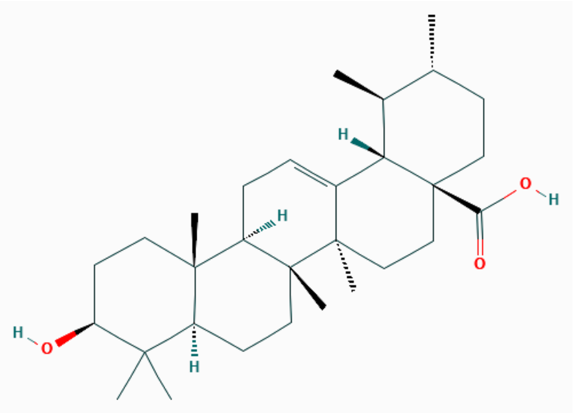	

Corosolic acid	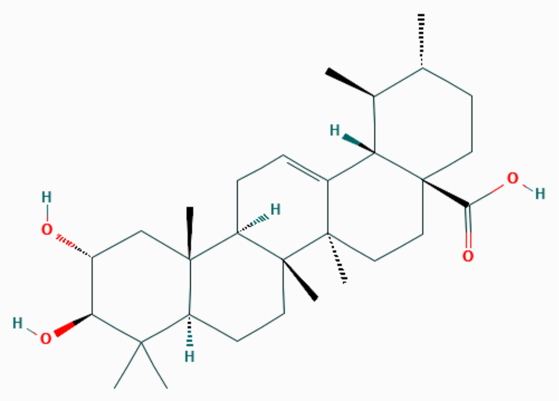
Esculentic acid	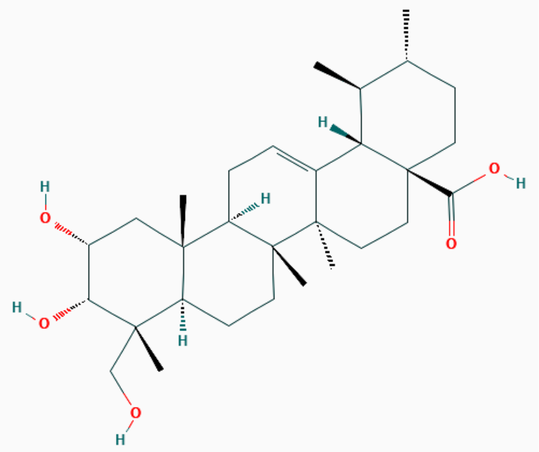
Lupeol	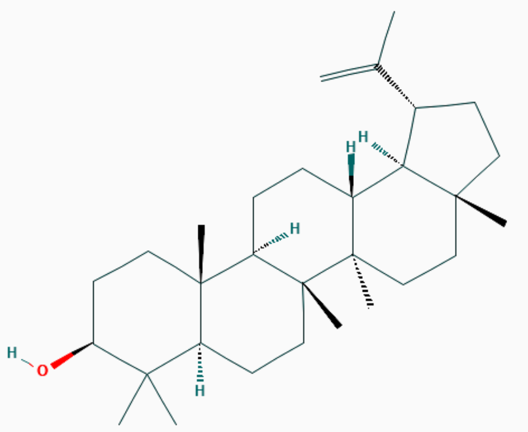

Lupenone	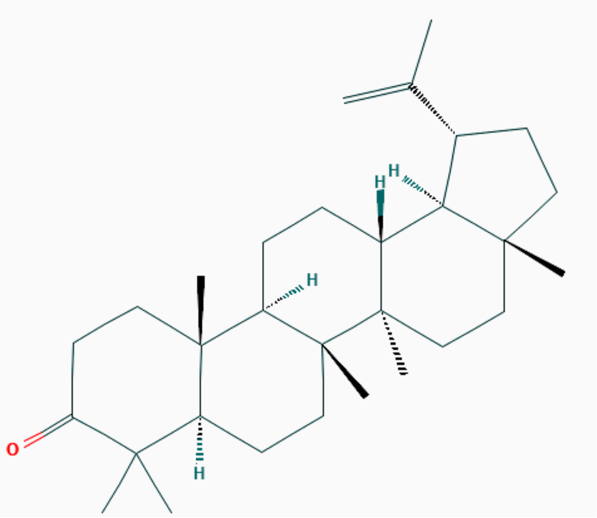
Betulinic acid	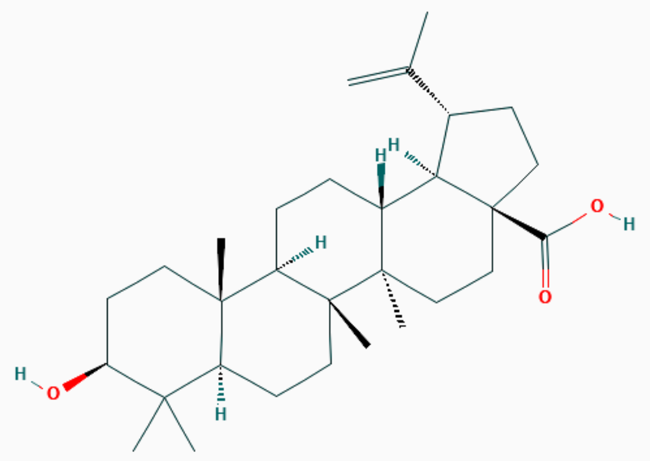
5α,8α epidioxyergosta-6,22-dien-3β-ol	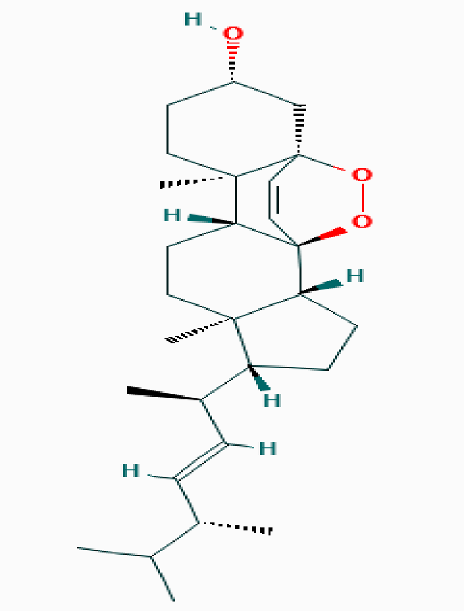

Cerevisterol	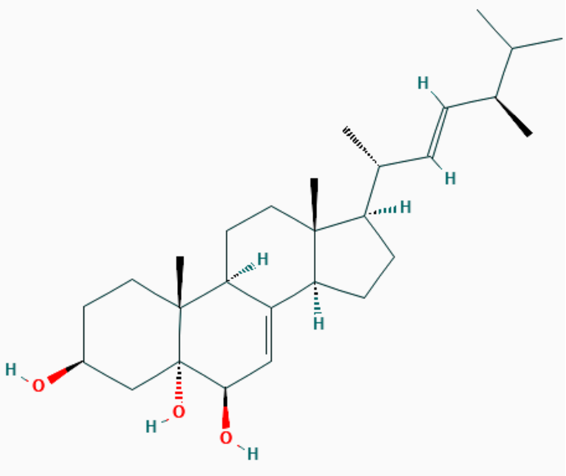
8,9-epoxyergosta-5,22-dien-3β,15-diol	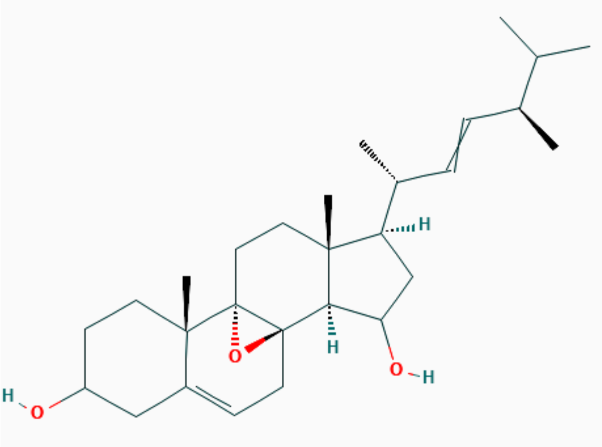
Ergosta-7,22-dien-3β-ol	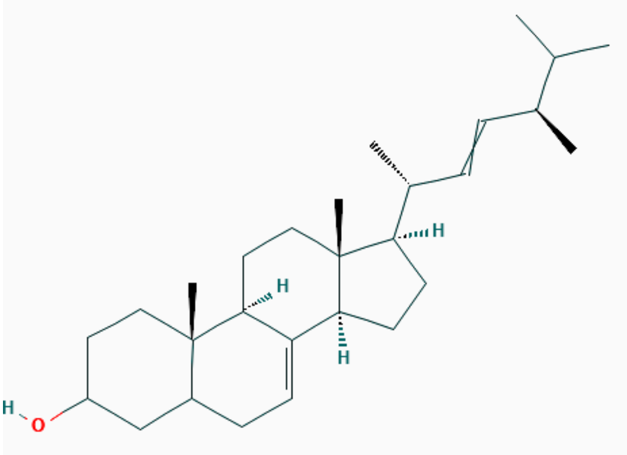

dihydrouridine	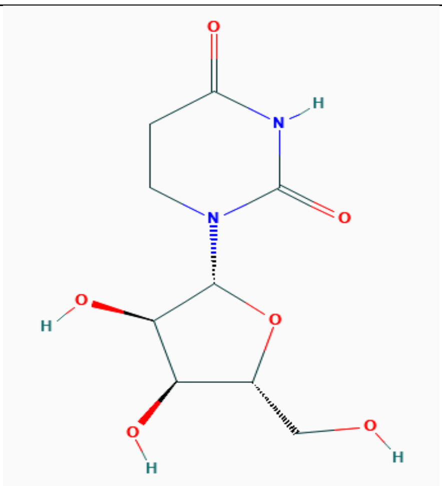
Deoxyuridine	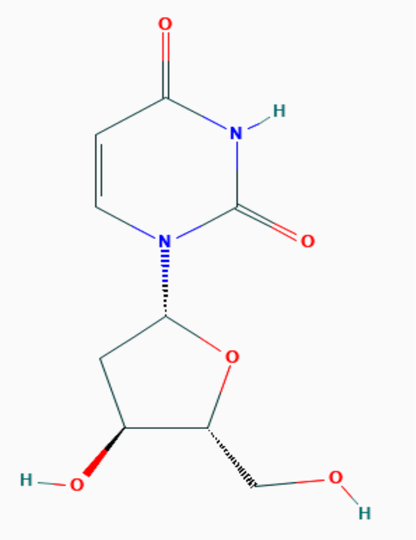
Vanillic acid	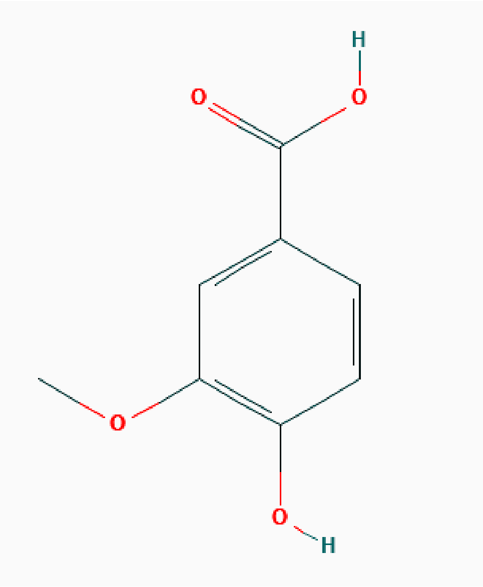

ethyl-β-d glucopyranoside	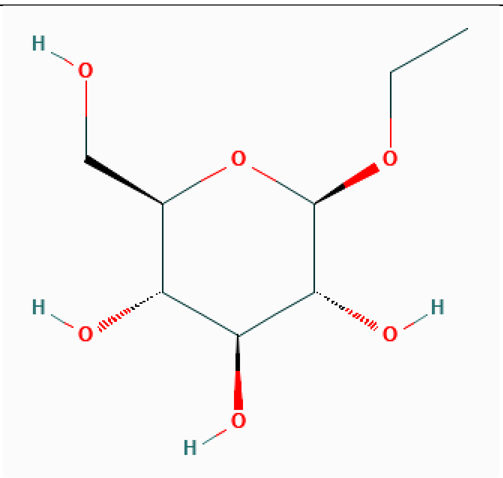
α-nigerose	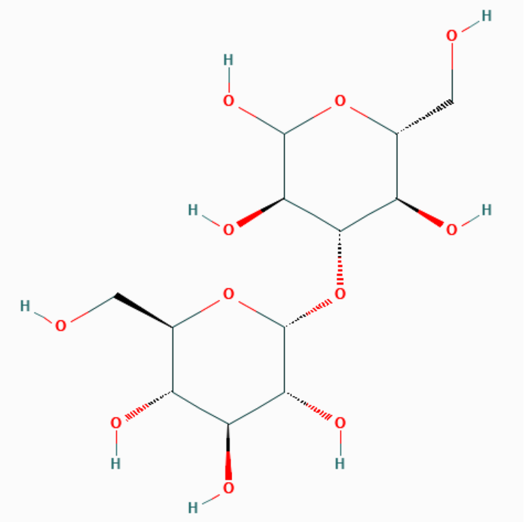
Trehalose	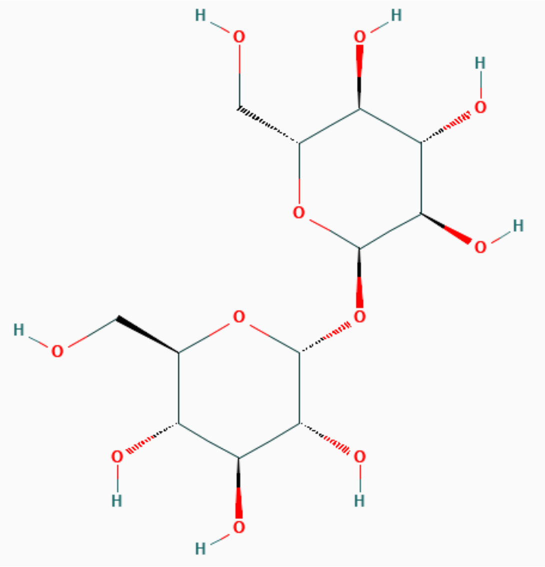

5-methoxyfurfural	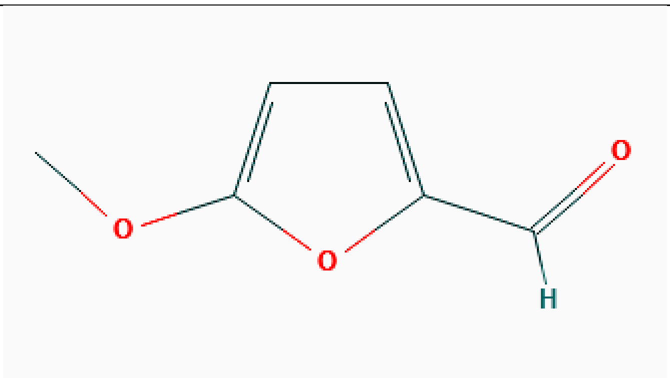

Table S2. Phenolic compounds of Trametes Versicolor identified through TLC technique [25]

Compound	Molecular formula	Molecular Structure	Nature of compound
Ergosterol	$C_{28}H_{44}O$	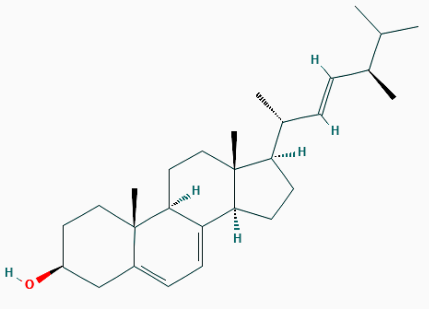	white amorphous powder
Ergosterol peroxide	$C_{28}H_{44}O_3$	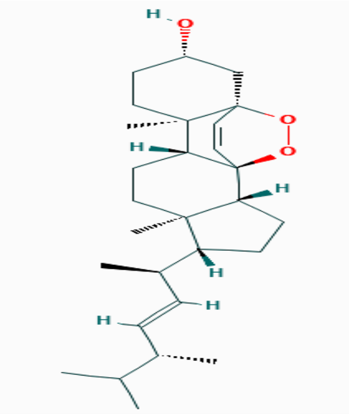	White crystalline
Trilinolein	$C_{57}H_{98}O_6$	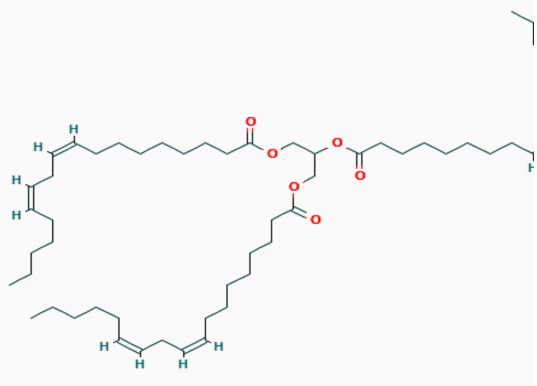	Yellow and oily

Ergosta-7,22-dien-3β-ol	$C_{28}H_{46}O$	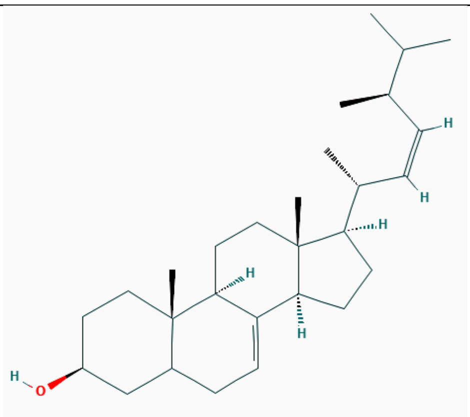	Colorless crystals
Betulinic Acid	$C_{30}H_{48}O_3$	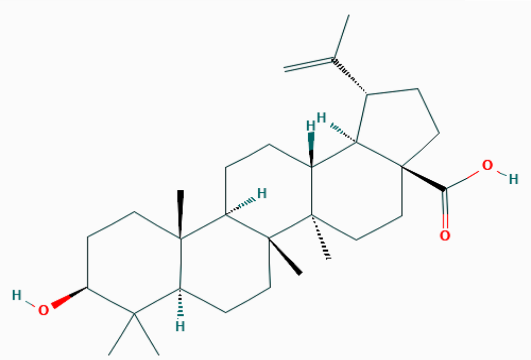	white crystalline solid
Betulin	$C_{30}H_{50}O_2$	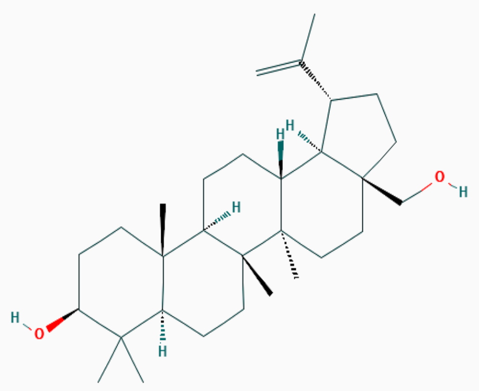	white crystalline solid

Table S3. Flavoring compounds isolated from Trametes versicolor by GC-MS/MS-O method [76]

Compound isolated	Molecular structure	Odor
ethyl 2-methylpropanoate	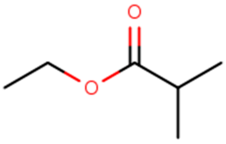	Fruity
3-methylbutanal	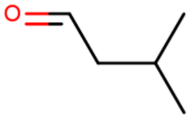	Fruity
2,3-butanedione	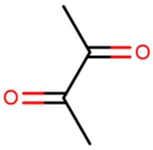	Buttery

methyl 2- methylbutanoate	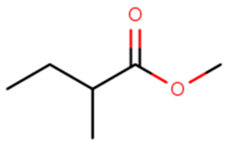	Fruity
ethyl butanoate	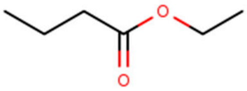	Fruity
ethyl 2- methylbutanoate	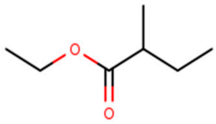	Fruity
hexanal	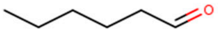	Fruity
2-pentanol	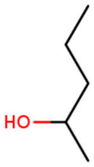	Fruity
2-methylbutanol	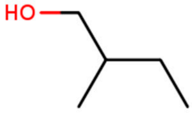	Fresh
3-methylbutanol	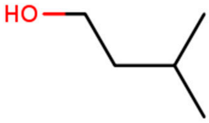	Sourish
1-octen-3-one	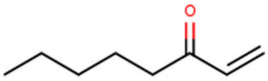	Mushroom
2,5-dimethylpyrazine	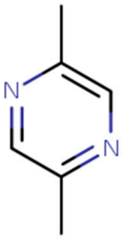	Baked nutty
Acetic acid	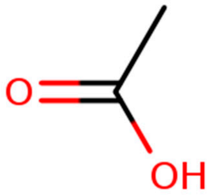	Sour vinegar
methional	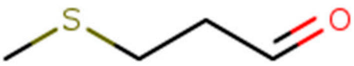	Boiled potato

benzaldehyde	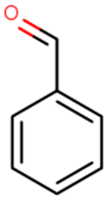	Almond, cereal
linalool	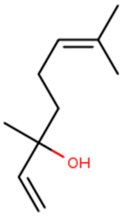	Floral
2-methylpropanoic acid	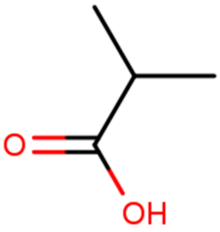	Stinky sour
2-phenylacetaldehyde	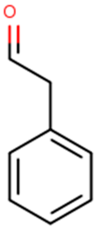	Floral
2-acetylthiazole	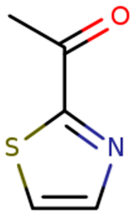	Sweet, toasted
2-methylpropanoic acid	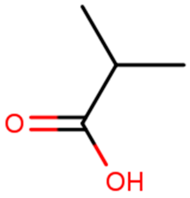	Stinky sour
2-phenylacetaldehyde	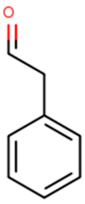	Floral
2-acetylthiazole	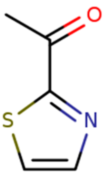	Sweet, toasted

2-methylbutanoic acid	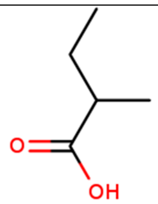	Cheesy
3-methylbutanoic acid	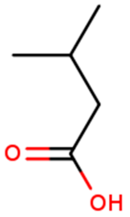	Sour, stinky
β-damascenone	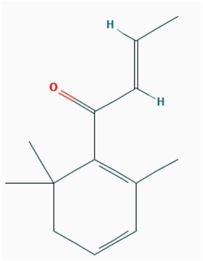	Fruity like pear
2-phenylethanol	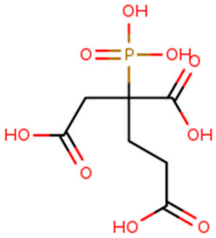	Rose
maltol	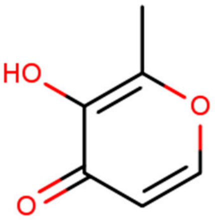	Caramel
2-acetylpyrrole	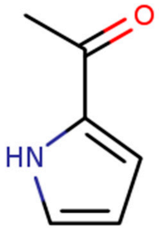	Sweet, burnt
γ-nonalactone	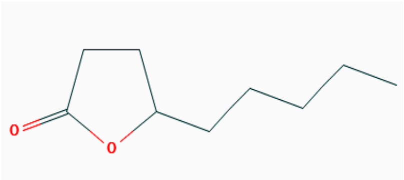	caramel
